# Therapeutic Effect of Losartan, an Angiotensin II Type 1 Receptor Antagonist, on CCl_4_-Induced Skeletal Muscle Injury

**DOI:** 10.3390/ijms17020227

**Published:** 2016-02-08

**Authors:** Ok-Kyung Hwang, Jin-Kyu Park, Eun-Joo Lee, Eun-Mi Lee, Ah-Young Kim, Kyu-Shik Jeong

**Affiliations:** 1 Department of Pathology, College of Veterinary Medicine, Kyungpook National University, Daegu 702-701, Korea; c3602@naver.com (O.-K.H.); jinkyu820@knu.ac.kr (J.-K.P.); miffy525@hanmail.net (E.-J.L.); nikeun@hanmail.net (E.-M.L.); pretersensual@hanmail.net (A.-Y.K.); 2 Stem Cell Therapeutic Research Institute, Kyungpook National University, Daegu 702-701, Korea

**Keywords:** CCl_4_, losartan, skeletal muscle, TGF-β1

## Abstract

TGF-β1 is known to inhibit muscle regeneration after muscle injury. However, it is unknown if high systemic levels of TGF-β can affect the muscle regeneration process. In the present study, we demonstrated the effect of a CCl_4_ intra-peritoneal injection and losartan (an angiotensin II type 1 receptor antagonist) on skeletal muscle (gastrocnemius muscle) injury and regeneration. Male C57BL/6 mice were grouped randomly as follows: control (*n* = 7), CCl_4_-treatment group (*n* = 7), and CCl_4_ + losartan treatment group (*n* = 7). After CCl_4_ treatment for a 16-week period, the animals were sacrificed and analyzed. The expression of dystrophin significantly decreased in the muscle tissues of the control group, as compared with that of the CCl_4_ + losartan group (*p* < 0.01). p(phospho)-Smad2/3 expression significantly increased in the muscles of the control group compared to that in the CCl_4_ + losartan group (*p* < 0.01). The expressions of Pax7, MyoD, and myogenin increased in skeletal muscles of the CCl_4_ + losartan group compared to the corresponding levels in the control group (*p* < 0.01). We hypothesize that systemically elevated TGF-β1 as a result of CCl_4_-induced liver injury causes skeletal muscle injury, while losartan promotes muscle repair from injury via blockade of TGF-β1 signaling.

## 1. Introduction

Carbon tetrachloride (CCl_4_) is a toxic chemical that is often employed to study the mechanisms of hepatotoxic effects associated with hepatic steatosis, fibrosis, and hepatocellular carcinogenicity [1]. CCl_4_ is mainly metabolized by cytochromes such as CYP2B1, CYP2B2, CYP2E1, and CYP3A to induce the formation of the trichloromethyl radical (CCl_3_) in the liver [2,3]_._ This radical can bind to various molecules including nucleic acids, proteins, and lipids, which leads to impaired lipid metabolism, potentially leading to steatosis [4]. Adduct production between CCl_3_ and DNA appears to initiate hepatocellular carcinoma [1]. Thus, CCl_4_ is well known to induce severe acute and chronic liver injury. However, TGF-β and reactive oxygen species (ROS), which are released from the liver in high amounts after CCl_4_ treatment [1,5], seem to induce secondary damage to other organs such as skeletal and cardiac muscle. To date, the effect of CCl_4_ on other organs excluding the liver is not well understood.

TGF-β1 belongs to a family of cytokines that transduce their signals through the Smad intracellular signaling cascade [6,7]. Olson *et al.* [8] previously reported that TGF-β1 impairs myocyte differentiation during myogenesis. TGF-β1 is a key factor in the differentiation of myoblasts into fibrotic cells [9], and is also associated with the occurrence of muscular fibrosis in patients having Duchenne’s muscular dystrophy, a degenerative muscle disease, and chronic inflammatory muscle disease [10]. The canonical TGF-β1 pathway is thought to affect various factors regulating myogenesis.

Studies of the angiotensin II (AT-II) receptor blockade of the renin-angiotensin system (RAS) led to the discovery of angiotensin-converting-enzyme (ACE) inhibitors [11]. ACE inhibitors are known to be effective in the treatment of hypertension, however, they are also associated with a high incidence of coughing and other adverse effects [11]. Several clinical studies have demonstrated that AT-II receptor antagonists such as candesartan, eprosartan, losartan, irbesartan, tasosartan, telmisartan, and valsartan are as effective as ACE inhibitors for the treatment of hypertension; furthermore, they induce fewer adverse effects [11,12]. The treatment of patients with AT-II receptor blockade results in ameliorated muscle wasting and reduced amounts of adipose tissue in their skeletal muscle tissues [13]. These positive effects may be mediated by direct action on the skeletal muscle. The AT-II receptor blockade is also known to inhibit the action of TGF-β1, which is also involved in the impairment of muscle regeneration in chronic myopathic disease [7]. Overall, the AT-II receptor blockade seems to attenuate TGF-β signaling in skeletal muscle.

The principal myogenic stem cell is the satellite cell, located between the plasma membrane and the basal lamina of muscle myofibers [14,15]. When stimulated by muscle damage, satellite cells become activated and start to proliferate profusely; they ultimately fuse with existing muscle fibers or fuse to form new myofibers [15]. An important component for the regeneration of skeletal muscle is to maintain the population of satellite cells via self-renewal, which is accomplished through proliferation and the activating signals of Pax7 [15]. MyoD (myoblast marker) and myogenin (fusion markers for myofibers) are two important myogenic regulatory factors [16] that function as transcription regulatory proteins by binding to the enhancer regions of numerous muscle-specific genes [16]. MyoD and myogenin play a key role during embryonic and neonatal myogenesis, and an increase in the expression of MyoD and myogenin in the skeletal muscle of aged animals has been previously observed [16,17,18].

In this study, we show for the first time that skeletal muscle is impaired by the production of TGF-β1 as a result of CCl_4_-induced chronic liver injury, and that the blockade of angiotensin II type 1 receptor by losartan treatment is protective against TGF-β1-induced skeletal muscle injury.

## 2. Results

### 2.1. Chronic CCl_4_ Injection Induces Muscle Damage

**Figure 1:**
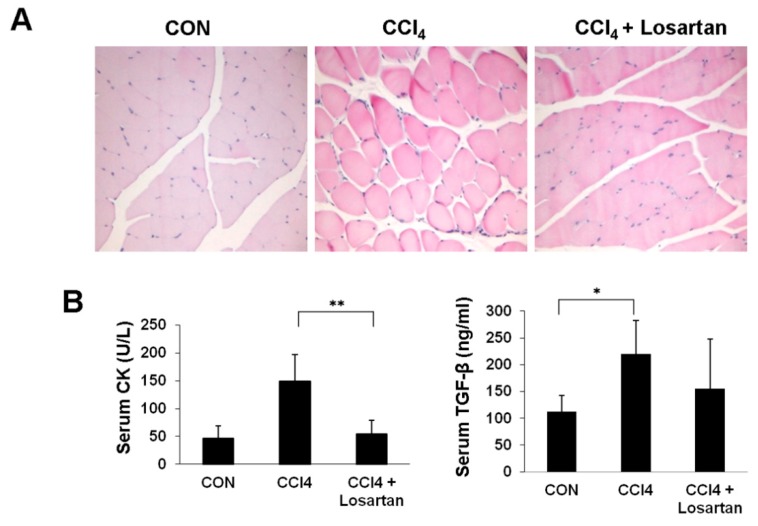
Histopathological change in skeletal muscle after CCl_4_ injection for 16 weeks (**A**); Serum level of CK (creatine kinase) (U/L) and TGF-β1 (ng/mL) (**B**). Original magnifications: ×200. Data is shown as mean ± SD (* *p* < 0.05, ** *p* < 0.01).

Chronic intraperitoneal administration of CCl_4_ induces liver injury, and in turn leads to skeletal muscle injury. The control group had intact normal muscle morphology, while the CCl_4_-treated group showed muscular atrophy and sarcopenia phenomena (Figure 1A). In contrast to the CCl_4_-treated group, there was a prominent decrease in the degree of muscular atrophy in the CCl_4_ + losartan-treated group (Figure 1A). Creatine kinase (CK), an enzyme released from damaged skeletal muscles into the blood, is potentially elevated in the serum when a muscle disorder is present. Accordingly, in the serum biochemical analysis, the levels of CK (Figure 1B) and TGF-β1 (Figure 1B) in the serum were observed to be highest in the CCl_4_-treated group, while their levels were attenuated in the CCl_4_ + losartan-treated group. A similar pattern was observed between CK and TGF-β1 levels.

### 2.2. Expression of Dystrophin in Skeletal Muscle

**Figure 2:**
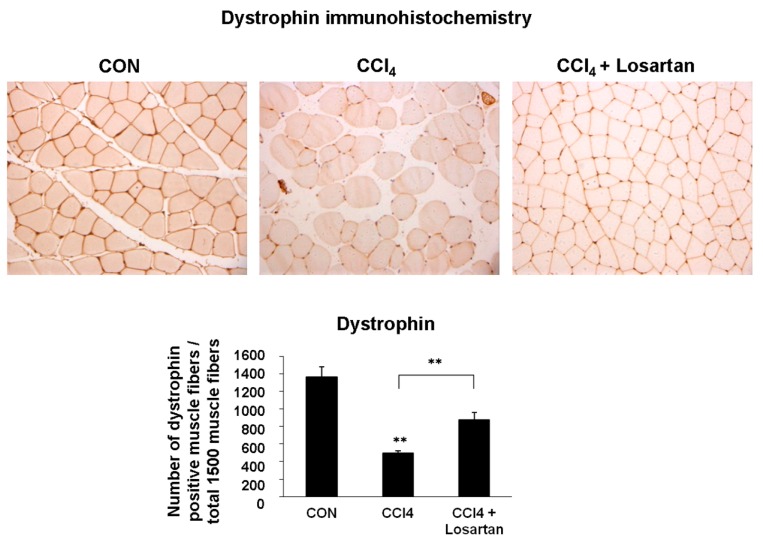
Immunohistochemical analysis of dystrophin (brown) with methyl green counter staining (green nuclei). Original magnifications: ×200. Estimation of the dystrophin expression level was quantified by counting the dystrophin-positive cells in three fields. Data is shown as mean ± SD (** *p* < 0.01).

Dystrophin is a subsarcolemmal actin-binding protein. It links the actin cytoskeleton and the extracellular matrix with a glycoprotein complex. Its absence is correlated with the fatal muscle-wasting disease, Duchenne muscular dystrophy [19]. In immunohistochemistry, the structural muscle protein dystrophin was expressed on the sarcolemma of the skeletal muscle. Dystrophin expression was significantly decreased in the CCl_4_-treated group compared with the CCl_4_ + losartan-treated group (*p* < 0.01) (Figure 2), indicating protection of the muscle myofiber by losartan.

### 2.3. Expression of p(phospho)-Smad2/3 and p-Smad3 in Skeletal Muscle

**Figure 3:**
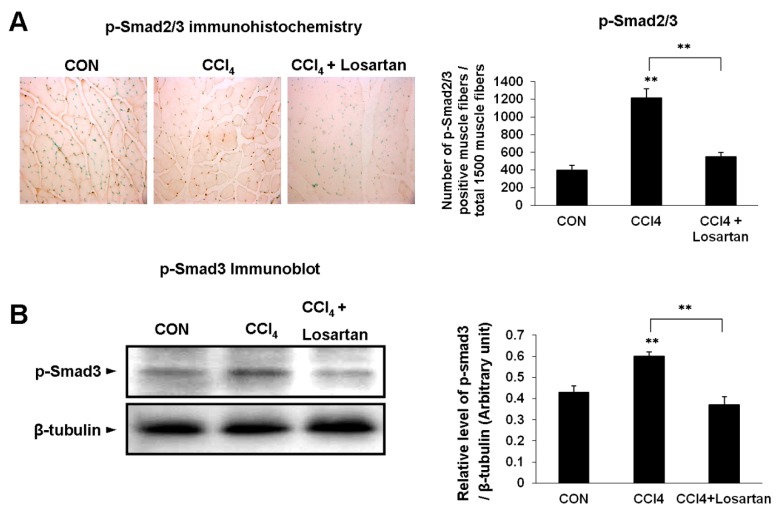
Immunohistochemical analysis of nuclear p(phospho)-Smad2/3 (brown nuclei) with methyl green counter staining (green nuclei). Original magnifications: ×200 (**A**); The positivity of each antigen in the muscle fibers was expressed as a distribution of the percentage of the total 1500 myofibers analyzed on the ×400 field. Data is shown as mean ± SD (** *p* < 0.01). Immunoblotting of p-Smad2/3 (**B**). The graph represents the relative band density to β-tubulin. Data is shown as mean ± SD (** *p* < 0.01).

p(phospho)-Smad2/3 is an intracellular protein immediately downstream of TGF-β1 signaling, and is present in the nucleus of injured skeletal muscle. Immunohistochemistry for the CCl_4_-treated group indicated a stronger positive expression in the nucleus of the injured skeletal muscle than those of the CCl_4_ + losartan-treated group (Figure 3A). p-Smad3 was also detected in the immunoblot analysis, and was well-matched with immunohistochemical examination in mice (Figure 3B).

### 2.4. Expression of Pax7 in Skeletal Muscle

**Figure 4:**
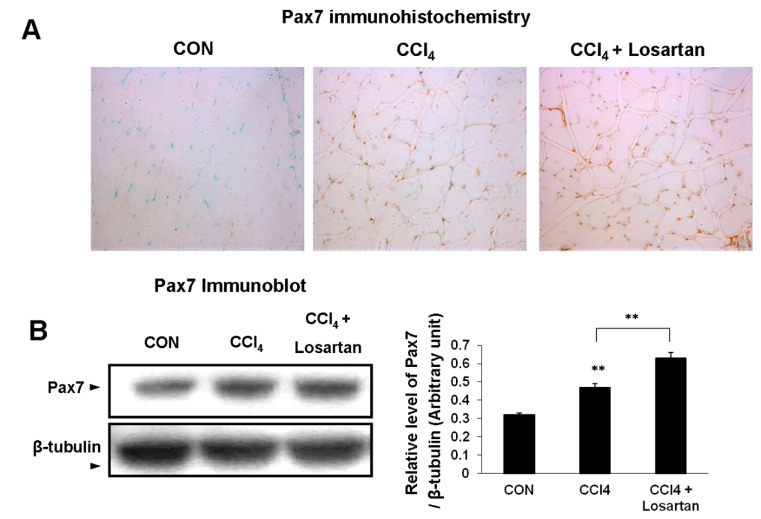
Immunohistochemical analysis of Pax7 (brown nuclei) with methyl green counter staining (green nuclei). Original magnifications: ×200 (**A**); Immunoblotting of Pax7 (**B**). The graph represents the relative band density to β-tubulin. Data is shown as mean ± SD (** *p* < 0.01).

When satellite cells are activated and proliferate, they express the transcription factor pax7, indicating self-renewal. The expression of pax7 was visualized by immunohistochemical analysis on skeletal muscle sections and immunoblot analysis in the skeletal muscle homogenates of mice. In the immunohistochemical examination, the expression level of pax7 was higher in the CCl_4_-treated group when compared with the control group (Figure 4A). The CCl_4_ + losartan-treated group showed a more significant increase than the CCl_4_-treated group (Figure 4A). The expression levels of the immunoblot and immunohistochemical analysis for pax7 were well matched (Figure 4B).

### 2.5. Expression of MyoD in Skeletal Muscle

**Figure 5:**
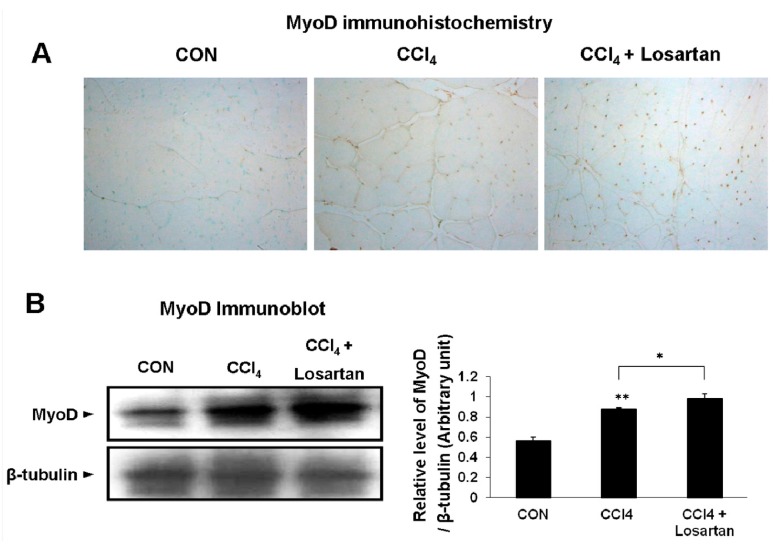
Immunohistochemical analysis of MyoD (brown) with methyl green counter staining (green nuclei). Original magnifications: ×200 (**A**); Immunoblotting of MyoD (**B**). The graph represents the relative band density to β-tubulin. Data is shown as mean ± SD (* *p* < 0.05, ** *p* < 0.01).

To investigate the quantity of myoD, both immunohistochemical examination and immunoblot analysis were performed. In the immunohistochemistry, the CCl_4_-treated group showed a more significant increase in myoD expression compared with the control group (Figure 5A). The CCl_4_ + losartan-treated group was observed to have the highest increase in myoD expression (Figure 5A). The immunoblot analysis for myoD was also detected and well matched with the immunohistochemical examination in mice (Figure 5B).

### 2.6. Expression of Myogenin in Skeletal Muscle

**Figure 6:**
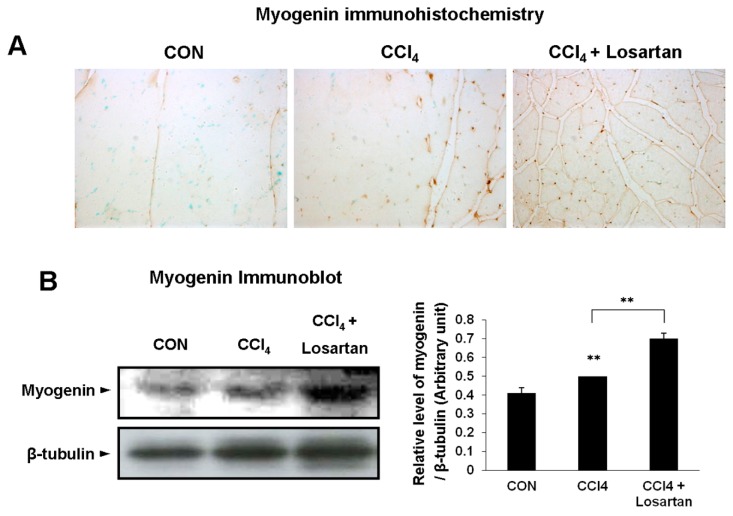
Immunohistochemical analysis of myogenin (brown), with methyl green counter staining (green nuclei). Original magnifications: ×200 (**A**); Immunoblotting of myogenin (**B**). Graph represents the relative band density to β-tubulin. Data is shown as mean ± SD (** *p* < 0.01).

In the immunohistochemistry, the expression level of myogenin was higher in the CCl_4_-treated group than in the control group (Figure 6A). The CCl_4_ + losartan-treated group had a more significant increase in the expression of myogenin than the CCl_4_-treated group (*p* < 0.01) (Figure 6A). The immunoblot analysis for myogenin was also detected and well matched with the immunohistochemical examination in mice (Figure 6B).

## 3. Discussion

CCl_4_ has been proven to be a highly useful experimental reagent in the study of certain types of hepatic damage [20,21,22]. CCl_4_ induces the release of tumor necrosis factor α (TNF-α), transforming growth factor α (TGF-α), transforming growth factor β (TGF-β), and nitric oxide (NO) from the affected cells, resulting in cell destruction or fibrosis. TNF-α pushes cells toward apoptosis, whereas the TGFs appear to direct them towards fibrosis [1]. TGF-β plays a key role in the fibrogenic response of skeletal muscle after CCl_4_ treatment [23,24,25,26]_._ We found that not only liver but also skeletal muscle is impaired by chronic CCl_4_ intoxication.

The CCl_4_-treated group had the highest level of serum CK and serum TGF-β1. This suggests that the skeletal muscle was damaged via elevated systemic circulation of TGF-β1 as a result of CCl_4_ injection. Several studies have reported that oxidative stress can injure the skeletal muscle [27,28]. Oxidative stress is involved in the pathogenesis of a number of chronic diseases including muscle wasting conditions such as muscle atrophy and age-dependent skeletal muscle wasting [27,29]. It also correlates with proinflammatory cytokines [27]. Cytokines stimulate peripheral neutrophils infiltrating tissues and further generating excess ROS (reactive oxygen species) [28]. Smith *et al.* [30] demonstrated that TGF-β expression in muscle tissues following skeletal muscle injury increased in rats, indicating that TGF-β is implicated in inflammatory processes. Several studies have shown that losartan, an antifibrotic agent directly antagonizing TGF-β1, improves the regeneration of injured skeletal muscle and inhibition of fibrous tissue deposits; it also appears to enhance myofiber regeneration [31,32,33,34,35]. In the same manner, we found that the losartan treatment protected the skeletal muscle by inhibiting TGF-β1 signaling. p-Smad2/3, an intracellular protein downstream of the TGF-β1 signaling pathway, was also significantly reduced in the CCl_4_ + losartan-treated group compared with the CCl_4_-treated group, as determined by immunohistochemistry and immunoblot, respectively.

The deficiency of dystrophin is associated with muscle degeneration. Duchenne muscular dystrophy (DMD), which occurs when dystrophin was depleted by a mutation of gene coding dystrophin on the X chromosome, induces muscle necrosis, damage, fibrosis, and weakness [36]. Interestingly, the expression of dystrophin was significantly higher in the CCl_4_ + losartan-treated group compared with that of the CCl_4_-treated group, as determined by immunohistochemistry. CCl_4_ administration can induce a decrease in the level of dystrophin, and the resulting atrophied skeletal muscle fiber which is similar to those found in DMD. Based on our present data, it is thought that these systemic levels of TGF-β may be associated with muscular atrophy, fibrosis, and the aging process in humans and animals.

Furthermore, markers of muscle stem cells such as Pax7, MyoD, and myogenin were increased in CCl_4_ + losartan-treated group. Muscle-specific stem cells (satellite cells) are very essential in generating new muscle after skeletal muscle injury [37,38]. Satellite cells are activated by not only pathological changes including muscle injury and degenerative disease but also physiological stimuli such as exercise [39]. In the present study, we also observed significantly increased Pax7 expression level after CCl_4_ administration and much higher level in CCl_4_ + losartan-treated group (Figure 4). Since Pax7 was previously shown to be expressed in proliferating satellite cells playing an important role in skeletal muscle regeneration [26], these data suggest that losartan improved self-renewal of satellite cells in injured muscle by inhibiting TGF-β1. Satellite cells were also known to play an important role in maintaining a population of myoblasts which can be differentiated into mature skeletal muscle fibers [39]. Myoblasts are able to fuse with existing myofibers to repair damaged muscle fibers, or alternatively fuse to each other to form new myofibers [40]. MyoD and myogenin play a critical role in the differentiation of satellite cells to the formation of myofibers [7,8]. In the present study, their expression patterns (Figure 5 and Figure 6) were very well-matched with those of Pax7 indicating that losartan promotes muscle regeneration after CCl_4_-induced muscle injury by enhancing muscle differentiation. Therefore, given that TGF-β1 signaling is related to the depression of muscle stem cells [41], losartan is thought to promote muscle regeneration by increasing the proliferation and differentiation of muscle stem cells to overcome the anti-myogenic effect of TGF-β1 [25,42].

To date, most previous studies have used CCl_4_ to induce liver injury. However, our experiments demonstrated that skeletal muscle can be injured by CCl_4_ injection, which suggests that systemically elevated circulating TGF-β1 produced by CCl_4_-induced liver injury can induce skeletal muscle injury. This was supported by the repair of muscle injury in the presence of losartan, an anti-TGF-β1 agent. Therefore, elevated TGF-β1 during chronic inflammation and the aging process can be thought to impair skeletal muscle. Previously, it was reported that blood TGF-β1 level was much higher in patients with severe chronic liver disease such as liver fibrosis, cirrhosis compared with controls [43], which means that these patients might have another risk for secondary skeletal muscle injury. Importantly, the present study provides very important evidence showing that increased TGF-β1 level by liver injury can be associated with skeletal muscle injury. Additionally, we also confirmed that losartan treatment is also protective against TGF-β1-induced skeletal muscle injury.

## 4. Experimental Section

### 4.1. Animals and Experimental Design

Twelve-week-old, male, C57BL/6 wild mice (*n* = 21) weighing 23–25 g were used in this study. Animals were maintained in a room with a temperature of 22 ± 2 °C and a relative humidity of 50% ± 10% with a 12-h light-dark cycle. They were divided into three groups: control group treated with olive oil, CCl_4_ intoxication group treated with olive oil containing 10% CCl_4_, and CCl_4_ + losartan group treated with olive oil containing 10% CCl_4_ and supplemented with a daily intake of losartan in drinking water (0.6 mg/mL). Average daily dose of losartan intake was 3 mg/5 mL/mouse. The animals were injected with intraperitoneal 10% CCl_4_ dissolved in 1 mL olive oil per kg body weight three times a week for up to 15 weeks. After 16 weeks, all the mice were sacrificed. We performed all animal experiments in compliance with the NIH (US) guidelines for the care and use of laboratory animals. All animal experiments were approved by Kyungpook National University Institutional Animal Care and Use Committee (IACUC).

### 4.2. Serum Biochemistry

Mice were anesthetized with ether and their blood collected to perform rapid serum biochemical analyses from the caudal vena cava. The blood was centrifuged at 3000 rpm for 15 min to separate the serum, which was immediately frozen until analysis. The serum creatine kinase (CK) level was measured using an ultraviolet method. The serum level of TGF-β1 was assayed using a TGF-β1 ELISA kit (R&D Systems Europe, Ltd., Abingdon, UK) according to the manufacturer’s instructions. The color product was measured at 450 nm using an ELISA reader (Tecan, Salzburg, Austria).

### 4.3. Histopathology and Immunohistochemistry

Samples of muscle tissue (gastrocnemius muscle) from each mouse were rapidly collected and fixed using 10% neutral buffered formalin and embedded using paraffin wax. Tissues sectioned at 4 µm were stained with hematoxylin and eosin (H&E). For immunohistochemistry, sections of muscle were deparaffinized in xylene, rehydrated through graded ethanol solutions, and washed in distilled water. Endogenous peroxidase activity was inhibited using 3% hydrogen peroxide in methanol for 30 min. The tissue sections were microwaved at 750 W for 10 min in a 10 mmol/L citrate buffer (pH 6.0). The tissue sections were washed with phosphate-buffered saline (PBS). The sections were immunostained with a primary antibody: monoclonal mouse anti-dystrophin antibody (diluted to 1:10) (Novocastra Laboratories, Newcastle Ltd., Newcastle, UK), polyclonal rabbit anti-p-Smd2/3 antibody (diluted to 1:800) (Santa Cruz Biotechnology, Santa Cruz, CA, USA), monoclonal mouse anti-Pax7 antibody (diluted to 1:1000) (Developmental Studies Hybridoma Bank, Tokyo, Japan), monoclonal mouse anti-MyoD antibody (diluted to 1:100) (Santa Cruz Biotechnology, Inc.), or monoclonal mouse anti-myogenin antibody (diluted to 1:200) (Santa Cruz Biotechnology, Inc.). The antigen-antibody complex was detected by using an avidin-biotin peroxidase complex solution with an ABC kit (Vector Laboratories, Burlingame, CA, USA) and followed by diaminobenzidine (DAB) as a chromogen. Sections were then rinsed in distilled water and counter-stained with Methyl green. The positivity of each antigen in the muscle fibers was expressed as a distribution of the percentage of the total 1500 myofibers analyzed.

### 4.4. Immunoblot Analysis

Snap-frozen muscle tissues were homogenized in RIPA buffer containing 1 mM sodium orthovanadate (Na_3_VO_4_), 50 mM NaF, and protease inhibitor (Roche, Mannheim, Germany). The tissue lysate was centrifuged for 10 min at 3000 rpm with temperature of 4 °C to remove debris with solid tissue components. Finally, the supernatant was centrifuged for 20 min at 14,000 rpm to get soluble cytosolic proteins. Protein concentration was measured using the Bradford method. Proteins were loaded on 10%–12% SDS-polyacrylamide gels for electrophoresis and were electro-transferred to PVDF membranes for immunoblotting (Schleicher & Schuell, Dassel, Germany) and non-specific binding was blocked with 3% bovine serum albumin in Tris-buffered saline (TBS). The membrane was immunoblotted with monoclonal mouse anti-Pax7 antibody (diluted to 1:1000) (Developmental Studies Hybridoma Bank, Tokyo, Japan), monoclonal mouse anti-MyoD antibody (diluted to 1:1000) (Santa Cruz Biotechnology, Inc.), and monoclonal mouse anti-myogenin antibody (diluted to 1:1000) (Santa Cruz Biotechnology, Inc.). Then, the membranes were incubated with horseradish peroxidase (HRP)-conjugated anti-mouse, rabbit, or rat IgG. Finally, target proteins were visualized by using the Super Signal West Dura Extended Duration Substrate (Pierce, Rockford, IL, USA) and exposed by medical X-ray film (Kodak, Tokyo, Japan).

### 4.5. Statistical Analysis

All data were shown as mean ± SD and determined for statistical significance according to the one-way analysis of variance (ANOVA). The value of statistical significance was set at *p* < 0.05.

## 5. Conclsions

Given that losartan has a protective effect on TGF-β1-induced skeletal muscle injury as well as liver fibrosis [25,44], it could be potentially used as a clinical therapeutic for skeletal muscle diseases induced by aging and other chronic diseases such as chronic liver fibrosis as well as chronic muscle atrophy, sarcopenia, and muscular fibrosis.
